# Current WHO recommendation to reduce free sugar intake from all sources to below 10% of daily energy intake for supporting overall health is not well supported by available evidence

**DOI:** 10.1093/ajcn/nqac084

**Published:** 2022-04-05

**Authors:** Rina Ruolin Yan, Chi Bun Chan, Jimmy Chun Yu Louie

**Affiliations:** School of Biological Sciences, Faculty of Science, The University of Hong Kong, Pokfulam, Hong Kong SAR, China; School of Biological Sciences, Faculty of Science, The University of Hong Kong, Pokfulam, Hong Kong SAR, China; School of Biological Sciences, Faculty of Science, The University of Hong Kong, Pokfulam, Hong Kong SAR, China

**Keywords:** free sugar, metabolic health, obesity, high fructose corn syrup, sucrose

## Abstract

Sugar is widely consumed over the world. Although the mainstream view is that high added or free sugar consumption leads to obesity and related metabolic diseases, controversies exist. This narrative review aims to highlight important findings and identify major limitations and gaps in the current body of evidence in relation to the effect of high sugar intakes on health. Previous animal studies have shown that high sucrose or fructose consumption causes insulin resistance in the liver and skeletal muscle and consequent hyperglycemia, mainly because of fructose-induced de novo hepatic lipogenesis. However, evidence from human observational studies and clinical trials has been inconsistent, where most if not all studies linking high sugar intake to obesity focused on sugar-sweetened beverages (SSBs), and studies focusing on sugars from solid foods yielded null findings. In our opinion, the substantial limitations in the current body of evidence, such as short study durations, use of supraphysiological doses of sugar or fructose alone in animal studies, and a lack of direct comparisons of the effects of solid compared with liquid sugars on health outcomes, as well as the lack of appropriate controls, seriously curtail the translatability of the findings to real-world situations. It is quite possible that “high” sugar consumption at normal dietary doses (e.g., 25% daily energy intake) per se—that is, the unique effect of sugar, especially in the solid form—may indeed not pose a health risk for individuals apart from the potential to reduce the overall dietary nutrient density, although newer evidence suggests “low” sugar intake (<5% daily energy intake) is just as likely to be associated with nutrient dilution. We argue the current public health recommendations to encourage the reduction of both solid and liquid forms of free sugar intake (e.g., sugar reformulation programs) should be revised due to the overextrapolation of results from SSBs studies.

## Introduction

Obesity, defined as having a BMI greater than or equal to 30 kg/m^2^, is a risk factor for various metabolic and endocrine abnormalities, such as hyperglycemia, hypertension, and dyslipidemia (1). The prevalence of obesity has increased dramatically in the past decades and is now considered an epidemic (1). High sugar consumption has been suggested to be obesogenic by inducing overeating and weight gain (2), and is considered a risk factor for chronic diseases, such as type 2 diabetes mellitus (T2DM) and cardiovascular disease (CVD) (3–5). In 2015, the WHO released a new recommendation of reducing the intake of free sugars, defined as sugar added to foods during production or cooking plus sugars found in honey, syrups, and fruit juices, to <10% of the daily energy intake, with a stricter target of <5% of daily energy intake for additional health benefits. The aim of this new recommendation on sugar was to reduce risks of all chronic diseases, especially for the prevention and control of obesity and dental caries (6).

The prevailing consensus among academics and public health practitioners is that high free sugar consumption is associated with ill health, as well as overweight and obesity, based on the concordance of available evidence from several sources (2, 3), as well as conclusions from systematic reviews and meta-analyses of available studies (7, 8). Nonetheless, it is a widely acknowledged fact that the publication of positive findings faces less resistance from journal reviewers and editors than that for null findings (9–11), and hence conclusions related to the free sugar–health relationship drawn from the literature may be somewhat affected by publication bias on an adverse association between the 2.

When examined carefully, it appears that most studies linking high free sugar intake to ill health focused on sugars from sugar-sweetened beverages (SSBs) (12–14), while studies examining free sugars from solid sources mostly reported null findings (15, 16). This has led to controversies regarding whether high free sugar consumption is detrimental to health, with some researchers suggesting that sugars are merely a source of calories, similar to proteins and fats (17). It also begs the question of whether the current public health recommendation to reduce free sugar intake from all sources (i.e., both solids and liquids) is well supported by the available evidence.

Therefore, in this review, we will highlight the limitations and problems in the current body of evidence, which may undermine the strength of study conclusions. We also propose that a rethink of whether all forms of free sugars are uniquely associated with ill health is warranted.

## Methods

### Criteria of human study selection

For human studies (observational studies and clinical trials) to be considered eligible for inclusion, they had to meet the following criteria: *1*) involved adult or children participants, who were either normal weight or overweight or obese at baseline; *2*) examined high-sugar diets in the form of solids, liquids, or both; and *3*) measured outcomes indicative of metabolic and endocrine health, such as body weight, fasting blood glucose and insulin levels, blood lipid levels, fat mass, and blood pressure. We imposed no restriction on the year of publication and included only articles in the English language. Reports such as unpublished manuscripts and conference abstracts were not included.

## Results

### Different metabolic consequences of intakes of glucose and fructose

Free sugars in the diet mostly come in the form of sucrose, which is digested into glucose and fructose in the gastrointestinal tract for absorption, as well as high-fructose corn syrup (HFCS), which contains a ∼1:1 ratio of glucose and fructose as monosaccharides (18). The metabolic fates of the 2 absorbed monosaccharides are different. The glucose metabolism is tightly regulated by insulin and hepatic energy statuses (19), where most postprandial glucose from the normal dietary intake will be metabolized in peripheral tissues, leaving little for storage as fat in the liver, thereby comprising a lower risk of developing chronic diseases, such as insulin resistance and T2DM, compared to fructose. Unlike glucose, the fructose metabolism is not regulated by insulin and hepatic energy needs, as the conversion to fructose 1-phosphate bypasses the key regulatory enzyme phosphofructose kinase-1 (20). Also, fructose does not stimulate insulin secretion (21), probably because of the absence of glucose transporter (GLUT) 5 in pancreatic β-cells (22). Thus, most fructose will be metabolized and stored by the liver, with little metabolism in peripheral tissues. It will also induce de novo lipogenesis (DNL) (23), resulting in hepatic fat accumulation (24), as well as insulin resistance and increased gluconeogenesis (19). Insulin resistance will further promote hepatic DNL, resulting in a vicious cycle that elevates VLDL production and secretion. Consequently, the plasma triglyceride (TG) concentration is even higher, leading to lipid accumulation in skeletal muscle, impaired insulin action, and whole-body insulin resistance (19). Additionally, the lack of insulin secretion after fructose ingestion also reduces leptin secretion by adipocytes (19), which may increase food intake, leading to weight gain and obesity (19, 22). It has also been proposed that high sugar consumption is detrimental for health due to its glycemic effects (25). However, only the glucose component of sugars has a high glycemic index (GI), while fructose has a low GI and sucrose has a moderate GI (26). Moreover, it has been suggested that the GIs of most high-sugar foods are low to moderate (27). Therefore, the glycemic effects of sugars per se should not have a major influence on cardiometabolic health. Overall, theoretically, excessive added or free sugar consumption could increase the risks of metabolic diseases through the direct actions of its constituent sugars and induction of weight gain indirectly (19), although whether this will happen at typical dietary doses remains controversial.

### Free sugars from solid compared with liquid foods: differential effects on health?

As mentioned earlier, much of the available evidence supporting weight gain in humans after high sugar consumption comes from studies focusing on SSBs (12–14), and few studies have directly compared the obesogenic effects of sugar in a solid compared with liquid form (19). This is important because studies have suggested that liquid sugar could elicit overeating, followed by incomplete compensation at subsequent meals, whereas solid sugar may not promote a positive energy balance (15, 16), despite most solid foods high in sugar also being high in energy density (28). Besides the difference in state (liquid vs. solid), there are also other differences which could contribute to the discrepancies in effects on metabolic health of solid compared with liquid sugars, such as the presence of caffeine (29), carbonation (30), and caramel colorings in some SSBs and in cola beverages (31). There have also been some observational studies conducted in children showing that SSB consumption leads to a higher risk of metabolic syndrome (MetSyn) than eating sugars in solid foods (32–37). For example, in a prospective cohort study conducted in African American and White children from the National Heart, Lung, and Blood Institute Growth and Health Study (*n* = 2021 at baseline; *n* = 5156 paired observations), after controlling for total energy intake, increased intake of liquid sugars was associated with an increase in waist circumference in all children over the 1-year follow-up period, whereas increased consumption of solid sugars was associated with an increased waist circumference in obese children only (32). In a prospective cohort study in Danish children from the European Youth Heart Study (*n* = 359), over the 6-year follow-up period, increased intake of liquid sugars predicted an increase in waist circumference and BMI, independent of energy intake, whereas intake of solid sugars did not (33). Intake of liquid but not solid sugars was associated with higher fasting glucose and insulin levels in Canadian Caucasian children in a prospective cohort study, as well as insulin resistance over the 2-year follow-up period after controlling for energy intake and physical activity (*n* = 630) (34). Higher intake of liquid but not solid sugars was linked with higher BMIs in girls (*n* = 1172) but not boys (*n* = 967) in Finnish children in a prospective cohort study, over the 21-year follow-up period (36). In Australian children, intake of liquid sugars was associated with a greater BMI over the 3.5-year follow-up period in a prospective cohort study (*n* = 158), compared with null findings in solid sugar (37).

A recent review of 7 epidemiological studies and 1 cross-over clinical trial (38) concluded that SSBs may be more likely to induce MetSyn than sugars in solid foods. The faster gastric emptying time for liquid sugars, and consequently the higher absorption of the fructose moiety, may lead to fat accumulation in the liver. Consumption of SSBs induces satiety less than solid sugar sources and is more likely to cause overeating or incomplete energy compensation at subsequent meals (39). This is important because the intestine may convert fructose to glucose when low concentrations are consumed, but fructose is transported to the liver more easily when consumed in high concentrations, such as from SSBs (40).

### Adverse health effects and proposed mechanisms of action: evidence from animal studies

Our concerns regarding the adverse health effects of high sugar consumption likely originated from animal studies. Mice or rats have been used to identify the culprits of potential detrimental health effects associated with high sugar intake (41, 42), as their genomes and organ systems are similar to those of humans, and they develop diseases in a comparable way to humans (43). However, mice and rats do differ from humans in the intermediary metabolism (44), which may undermine the translatability of rodent findings to advance human health (45). Therefore, conclusions from animal studies should be interpreted with caution.

Sucrose or fructose feeding of supraphysiological doses in both solid and liquid forms has been shown to induce insulin resistance, glucose intolerance, hyperglycemia, and hypertriglyceridemia in animals, mostly over the short term (**Table 1**) (46–51). For example, feeding rats with a high-sucrose diet (69% daily energy intake of a 74-kcal diet) for 4 weeks led to insulin resistance in the liver, compared to an isocaloric high-starch diet (*n* = 55) (51). Administration of a high-sucrose diet [68% weight by weight (w/w)] in rats for 1, 2, 5, or 8 weeks significantly impaired insulin action in the liver and muscle, and increased serum TG concentrations, compared with the starch control diet, which may be associated with insulin resistance (*n* = 8–10 per group per time point) (49). In another study, rats fed a 60% w/w fructose diet developed hyperglycemia and hyperinsulinemia when compared with the control group in 8 weeks (*n* = 24) (52).

**TABLE 1:** Summary of animal studies examining the effects of high sugar consumption on metabolic health^1^

Reference, year	Animals used	Study duration	Dietary intervention	Main findings
Asghar et al., 2016 (148)	Female C57BL/6J mice, unspecified number	6 wk (pregnancy and lactation)	HFrD (60%w/w fructose) vs. standard rodent chow (3% sucrose), ad libitum	HFrD resulted in placental insufficiency, and higher fetal serum glucose and TG
				HFrD also induced higher placental uric acid level, and activities of AMP deaminase and xanthine oxidase
Huang et al., 2004 (52)	24 male Sprague-Dawley rats	8 wk	HFrD (60% w/w) vs. standard rodent chow, ad libitum	Compared with standard rodent chow, the HFrD resulted in higher plasma glucose at the end of a 2-hour glucose challenge, and higher plasma leptin as well as fasting insulin and TG level
Kanarek and Orthen-Gambill, 1982 (46)	35 male Sprague-Dawley rats	50 d	Standard lab chow alone vs. standard lab chow plus 32% w/w glucose or fructose or sucrose solutions or granulated sucrose	Reduced glucose tolerance was observed in high-fructose and -sucrose groups vs. glucose groupThe high-fructose group also had significantly higher TG concentrations compared with the high-sucrose and high-glucose groups.
			
Lee et al., 2020 (47)	40 male C57BL/6 mice	13 wk	HFD vs. normal chow diet, each supplemented with water or SSB	The SSB-treated groups had significantly higher fasting glucose levels, as well as larger hepatic lipid droplets and adipocyte sizes compared with the control group. Expression of genes related to hepatic and adipose tissue inflammation also increased in the SSB group
Lombardo et al., 1996 (48)	16 male Wistar rats	30 wk	SRD (63% w/w) vs. standard rat laboratory chow, ad libitum	Compared with the control diet, the sucrose-rich diet resulted in higher blood glucose and TG levels, insulin resistance, and lower insulin secretion
Pagliassotti et al., 1996 (49)	130 male Wistar rats	1, 2, 5, or 8 wk	Semipurified starch diet (0% daily energy intake from sugar) vs. high-sucrose (68% w/w) diet, fed 95% of average food intake	The high-sucrose diet resulted in insulin resistance first in the liver, then in the muscle, which may be related to higher TG levels in these organs
Ruff et al., 2013 (50)	98 female and 58 male wild-derived mice	26 wk of dietary exposure	High-fructose and glucose diet (1:1 mixture providing 25% daily energy intake) vs. control diet (0% daily energy intake from sugars), ad libitum	The high-sugar diet resulted in 1.97 times higher death rates, as well as a 1.42 times lower glucose clearance rate, in female mice compared with the control diet, but no such effect was seen in male mice
				Male mice fed the high-sugar diet, however, controlled 26% less territory and produced 25.3% fewer offspring
				No effect on body weight was observed in either sex
Storlien et al., 1988 (51)	55 male Wistar rats	4 wk	Starch diet (0% daily energy intake from sugar) vs. SRD (69% daily energy intake), at 74 kcal/d	The SRD resulted in impairment in whole-body glucose disposal, due mainly to impairment in hepatic insulin action. However, it did not affect body fat accumulation

1 HFD, high-fat diet; HFrD, high-fructose diet; SSB, sugar-sweetened beverage; SRD, sucrose-rich diet; TG, triglyceride; w/w, weight/weight.

Results from longer-term studies on sugar in solid foods are similar. For example, rats that consumed a 63% w/w high-sucrose diet for 30 weeks developed hyperglycemia, hypertriglyceridemia, and insulin resistance, compared to the control group fed on an isocaloric high-starch diet (*n* = 16) (48). Interestingly, insulin secretion was not increased in the presence of pancreatic hypertrophy and hyperplasia, and there was also some β-cell derangement (48). Ruff et al. (50) showed that in wild-type mice, high sugar consumption (at 25% daily energy intake) for 26 weeks resulted in increased mortality in females (*n* = 98) and decreased controlled territories and reproductive success in males (*n* = 58), in addition to reducing glucose tolerance and increasing fasting cholesterol level in both sexes.

The effects of high sugar consumption in the liquid form on top of the standard lab chow diet were examined in 2 studies (46, 47). In 1 study, feeding 32% w/w fructose or sucrose solutions in addition to the standard lab chow to rats for 50 days led to reduced glucose tolerance, and significantly a higher TG concentration was also observed in those given a 32% w/w fructose solution, compared to rats given a 32% w/w glucose solution (46). Similarly, Lee et al. (47) also showed that supplementation of the standard lab chow diet with SSBs resulted in significantly higher fasting glucose levels, as well as accumulation of lipids in the liver. Expression of inflammatory genes in the liver and adipose tissues also increased (*n* = 40).

Overall, feeding excessive sugar (fructose or sucrose) to mice or rats, whether in solid or liquid form, could cause reduced competitive ability and metabolic abnormalities, including insulin resistance, hyperglycemia, and hypertriglyceridemia. These health effects are likely associated with the development of obesity.

#### Limitations of previous animal studies

While conclusions from animal studies generally support the adverse health effects of high sugar consumption, caution should be exercised in interpreting and translating the results, as several major limitations exist, which might explain why all studies, regardless of whether solid or liquid sugars were examined, found negative health effects of high sucrose or fructose consumption.

First, some studies used fructose alone as the treatment. However, fructose is rarely consumed alone in the human diet. Instead, it almost always coexists together with glucose in the form of sucrose or HFCS. Since the metabolism of pure fructose and its associated health consequences is different from when fructose is consumed as part of sucrose or consumed with glucose (as in HFCS) (41), it is a far reach to translate the conclusions related to excessive pure fructose consumption in rodents into the human situation. Also, most animal studies failed to include a control group where only glucose was consumed; therefore, it is unknown whether the adverse health effects observed are due to the high monosaccharide (fructose) consumption per se or to the energy supplied by fructose specifically (41, 42, 53).

Second, the majority of studies examined the health impacts of supraphysiological doses of sugars (typically >50% of the daily energy intake). These studies were designed to induce pronounced metabolic impairments in a short period, to investigate the mechanisms of action in laboratory animals. However, results obtained from such studies bear little resemblance to actual human consumption levels (54). Third, in designing the control diet, most studies opted to replace all sugars with starch, which is unrealistic and irrelevant to humans, as we rarely consume a diet devoid of sugars. On average, adults consume between 7% and 12% of their daily energy intake from added sugars (55). Fourth, no studies so far have directly compared the effects of solid compared with liquid sugars on metabolic and endocrine health in rodents, which makes it difficult to draw firm conclusions regarding the potential differences in their effects on metabolic and endocrine health. Last, while some rodent studies lasted more than 20 weeks, which covers a substantial period of a rodent's life span, most studies were conducted over a short period and rarely lasted longer than 6 to 8 weeks, thus impairing translatability into humans.

### Studies on high sugar consumption and metabolic or endocrinic disturbances in humans

#### Evidence from observational studies

Unlike in animal studies, there is great heterogeneity in the conclusions from observational studies in humans, with some supporting an association between high SSBs or sugar consumption and the development of metabolic diseases, while others report null findings (**Tables 2** and **3**). This might be due to differences in study designs, populations of interest, and the forms of sugar examined (e.g., SSBs vs. solid sugar). Also, many observational studies collect data via self-reporting of the participants: for example, from FFQs, dietary record, and dietary recalls (56, 57). Self-reported dietary data are often regarded as being unreliable, as they may be affected by selective recall and reverse causation. Differences in confounding factors across observational studies are also a concern, and they affect the abiltabity to synthesize evidence from various studies. Nonetheless, based on the Bradford Hill criteria, causality can be assumed only between SSB intakes and cardiometabolic disease risks, as most if not all studies showed consistent results; however, no causality can be assumed between total, added, and free sugar intakes and health outcomes, as results are largely inconsistent. Furthermore, it has been proposed that the Bradford Hill criteria should be adapted to the evolving nature of research to promote multidisciplinary research and data integration frameworks (58). Caution should therefore be exercised in interpreting findings from observational studies.

**TABLE 2:** Summary of observational studies examining the association between high SSB consumption on metabolic parameters and chronic diseases^1^

Reference, year	Subjects	Study duration	Dietary comparator	Main findings
Berkey et al., 2004 (112)	11,755 adolescents aged 9–14 y from the US Growing Up Today study (43.1% boys)	3 y	Consumption of SSBs	Before adjustment for total energy intake, consumption of SSBs was associated with increase in BMI in the corresponding year (boys: +0.03 kg/m^2^ per daily serving, *P* = 0.04; girls: +0.02 kg/m^2^, *P* = 0.096), compared with nondrinkers. Children who increased consumption by ≥2 servings/d from the prior year gained weight [boys: +0.14 kg (*P* = 0.01); girls: +0.10 kg (*P* = 0.046)], compared with those with unchanged intakes
				After adjustment for total energy intake, the effects were not significant
de Koning et al, 2012 (81)	42,883 males aged 40–75 y in the Health Professionals Follow-Up Study	22 y	SSB consumption (never vs. 2/mo vs. 1–3/wk vs. 3.7/wk to 9/d)	Higher SSB consumption was associated with increased risks of CHD (RR for never vs. 3.7/wk to 9/d: 1.18; 95%CI, 1.06–1.31; *P*_trend_ < 0.01 after adjustment for confounders)
				An increase in every serving of SSB per day was also associated with 12.7 (95% CI, 4.2–21.2) mg/dL higher TG (*P* = 0.01), 1.87 (95% CI, 1.03–2.70) mg/dL lower HDL (*P* < 0.01), 0.12 (95% CI, 0.02–0.23) mg/L higher CRP (*P* = 0.02), 0.16 (95% CI, 0.03–1.65) pg/mL higher IL-6 (*P* = 0.02), and 796 (95% CI, 149–1442) pg/mL lower leptin (*P* = 0.02)
den Biggelaar et al., 2020 (149)	2240 middle-aged subjects (mean ± SD age, 59.5 ± 8.1 y; 50.4% male)	NA (cross-sectional study)	Non-consumers vs. moderate or daily SSB consumers	No statistically significant difference in β-cell glucose sensitivity and potentiation factor, C-peptidogenic index, overall insulin secretion, and Matsuda index between nonconsumers vs. moderate or daily SSB consumers
Dhingra et al., 2007 (73)	Cross-sectional and longitudinal analyses of the Framingham Heart Study Cohort (6039 person- observations, 3470 in women; mean age 52.9 y)	3 y	Consumption of sugar-sweetened soft drinks	Cross-sectionally, consumption of ≥1 serving/d of sugar-sweetened soft drink was associated with increased prevalence of MetSyn (OR, 1.81; 95% CI, 1.28–2.56), compared to intake of <1 serving/wk
				Longitudinally, consumption of ≥1 serving/d was associated with increased incidence of MetSyn (OR, 1.62; 95% CI, 0.96–2.75), compared with infrequent drinkers (<1 serving/wk)
Duffey et al., 2010 (70)	2774 adults (mean ± SD age, 25.0 ± 3.6 y; females, 53.5% ± 0.8%) from the CARDIA study	20 y	Consumption of SSBs across quartiles	Higher SSB consumption was associated with increased risks of high WC (adjusted RR, 1.09; 95% CI, 1.04–1.14; *P*_trend_ < 0.001), high LDL cholesterol (adjusted RR, 1.18; 95% CI, 1.02–1.35; *P*_trend_ = 0.018), high TG (adjusted RR, 1.06; 95% CI, 1.01–1.13; *P*_trend_ = 0.033), and hypertension (adjusted RR, 1.06; 95% CI, 1.01–1.12; *P*_trend_ = 0.023) across quartiles
Eny et al., 2020 (90)	1778 preschool children aged 3–6 y (53.4% boys)	9 y	Consumption of sugar-containing beverage	An increase in every serving of sugar-containing beverage per day was associated with 0.02 (95% CI, 0.01–0.03) mmol/L lower HDL (*P* = 0.01) and 1.05 (95% CI, 1.01–1.10) mmol/L higher TG (*P* = 0.03) after adjustment for confounders
				No statistically significant association was observed between sugar-containing beverage consumption and blood glucose or systolic blood pressure
Fagherazzi et al., 2013 (61)	66,118 females (mean ± SD age, 52.6 ± 6.6 y) from the E3N cohort	14 y	SSB consumption (nonconsumer vs. <86 vs. 86–164 vs. 165–359 vs. >359 mL/wk)	Higher SSB consumption was associated with increased risks of T2DM (HR for nonconsumer vs. >359 mL/wk: 1.30; 95% CI, 1.02–1.66; *P*_trend_ = 0.021) after adjustment for confounders
Fung et al., 2009 (82)	88,520 females from the Nurses’ Health Study aged 34–59 y	24 y	SSB consumption in servings (<1/mo vs. 1–4/mo vs. 2–6/wk vs. 1 to <2/d vs. ≥2/d)	Higher consumption of SSBs was associated with increased risks of CHD (RR for <1/mo vs. ≥2/d: 1.35; 95% CI, 1.07–1.69; *P*_trend_ < 0.001)
Funtikova et al., 2015 (68)	2181 Spanish males and females aged 25–74 y	9 y	Changes in soft drink consumption (maintenance of no consumption vs. decrease in consumption vs. increase in consumption vs. maintained consumption)	100-kcal increase in soft drink consumption was associated with 1.1-cm increase in WC (*P* = 0.018), and higher soft drink consumption was associated increased odds of 10-year incidence of abdominal obesity (OR for no consumption vs. ≥200 ml/d, 1.77; 95% CI, 1.07–2.93)
Garduño-Alanís et al., 2020 (62)	5205 Russian adults aged 45–69 y (47% males) from the Health, Alcohol and Psychosocial factors in Eastern Europe cohort	3 y	Fruit juice or SSB consumers vs. nonconsumers	No statistically significant association between fruit juice consumption and unit change in BMI (drinkers vs. nondrinkers; OR, 0.92; 95% CI, 0.81–1.05; *P* = 0.203)SSB consumers had 26% (95% CI, 9%–45%) higher odds of having a 1-kg/m^2^ increase in their BMI in 3 years compared with nondrinkers
Hirahatake et al., 2019 (76)	4719 Black and White males and females aged 18–30 y at baseline from the CARDIA study (45.3% males)	30 y	SSB consumption in servings (none to ≤1/wk vs. 1 to ≤4/wk vs. 4 to ≤7/wk vs. 1–2/d vs. ≥2/d)	An increase in every serving/d of SSB was associated with a 6% (95% CI, 1%–10%) increase in the risk of T2DM (*P* = 0.009)
Harrington et al., 2020 (74)	1075 boys and girls aged 8–11 y (66.1% boys)	NA (cross-sectional analysis)	SSB consumption	Compared with normal-weight children, children with overweight or obesity had significantly higher intake of SSBs per day (383 vs. 315 mL). Also, children who consumed >200 mL per day of SSBs had a higher risk of overweight or obesity compared with those consuming <200 mL per day (OR, 1.8; 95% CI, 1.0–3.5)
Haslam et al., 2020 (88)	The FOS (*n* = 3146; mean ± SD age, 54.8 ± 9.8 y; 46.9% males) and Generation Three cohorts (*n* = 3584; mean ± SD age, 40.3 ± 8.8 y; 45.7% males)	12.5 y	SSB consumption from none or <1 serving per month to ≥6 servings/d	Compared with low consumption (<1 serving/mo), regular consumption (>1 serving/d) of SSBs was associated with a greater mean decrease in HDL cholesterol (β ± standard error, −1.6 ± 0.4 mg/dl; *P*_trend_ < 0.0001) and increase in TG concentrations (β ± standard error: 4.4 ± 2.2 mg/dl; *P*_trend_ = 0.003)
Imamura et al., 2019 (63)	27,662 adults from the EPIC-InterAct case-cohort study [mean ± SD age, 52.0 ± 9.0 y (38% males) and 56 ± 7.7 y (50% males) for randomly selected subcohort and ascertained cases of T2DM, respectively]	15 y	SSB consumption (per 250 g/d increase and 250 g/d vs. 0 g/d)	For every 250 g/d increase in SSB consumption, the risk of T2DM incidence increases by 18% (95% CI, 8%–28%)Comparing with nonconsumers (i.e., 0 g/d), those who consumed 250 g/d of SSB had a 7.4/10,000 person-years increase in T2DM rates
Janzi et al., 2020 (71)	25,877 adults aged 45–74 y (mean age, 57.8 y; 37.6% males) from the Malmö Diet and Cancer Study	19.5 y	Consumption of total added sugar and sugar-sweetened foods and beverages across categories	Added sugar intake > 20% daily energy intake was associated with increased risks of coronary events (HR, 1.39; 95% CI, 1.09–1.78) compared to the lowest intake category (<5% daily energy intake), and of stroke (HR, 1.31; 95% CI, 1.03–1.66), compared to 7.5%–10% daily energy intake
Lin et al., 2020 (89)	6856 adults from the NHANES (50.5% males)	3 y	SSB consumption [none vs. 1–350 (light) vs. 351–699 (medium) vs. ≥ 700 ml/d (heavy)]	Compared with nonconsumers, heavy SSB consumers had a 0.26 mg/l higher CRP level after adjusting for BMI. When taking into consideration the modifying effect of BMI, medium and heavy drinkers who were obese had 0.58 (*P* = 0.014) and 0.50 mg/l (*P* = 0.013) higher CRP levels than non-SSB drinkers, respectively
Ma et al., 2015 (64)	2634 participants of the Framingham Heart Study (47.5% males)	NA (cross-sectional analysis)	SSB consumption in servings (0–1/mo vs. 1/mo to <1/wk vs. 1/wk to <1/d vs. ≥1/d)	Higher SSB consumption was associated with increased odds of NAFLD (OR for 0–1/mo vs. ≥ 1/d, 1.56; 95% CI, 1.03–2.36; *P*_trend_ = 0.02) after adjustment for age, sex, dietary confounders, and smoking. The statistical significance was lost after additional adjustment for VAT
Malik et al., 2019 (84)	37,716 men from the Health Professional's Follow-up Study and 80,647 women from the Nurses’ Health Study	Health Professional's Follow-Up Study (28 y)Nurses’ Health Study (34 y)	SSB consumption (number of times of consuming a standard portion of foods and beverages; <1/mo vs. 1–4/mo vs. 2–6/wk vs. 1 to <2/d vs. ≥2/d)	Across categories, high SSB consumption was associated with higher risks of total mortality in a dose-response relationship [HRs of 1.00 (reference), 1.01 (95% CI, 0.98–1.04), 1.06 (95% CI, 1.03–1.09), 1.14 (95% CI, 1.09–1.19), and 1.21 (95% CI, 1.13–1.28) for consumption frequencies of <1/mo, 1–4/mo, 2–6/wk, 1 to <2/d and ≥2/d, respectively; *P* < 0.0001]
				High SSB consumption was also associated with increased risks of CVD mortality across categories [HRs of 1.00 (reference), 1.06 (95% CI, 1.00–1.12), 1.10 (95% CI, 1.04–1.17), 1.19 (95% CI, 1.08–1.31), 1.31 (95% CI, 1.15–1.50) for consumption frequencies of <1/mo, 1–4/mo, 2–6/wk, 1 to <2/d and ≥2/d, respectively; *P* < 0.0001]
O'Conner et al., 2018 (104)	9678 British adults (mean ± SD age, 47.8 ± 7.4 y; 46.6% males)	NA (cross-sectional analysis)	Sugar intake from liquid foods and solid foods (Q1: 0.5–8.0 vs. Q2: 8.0–10.4 vs. Q3: 10.4–12.6 vs. Q4: 12.6–15.5 vs. Q5: 15.5–46.4; % daily energy intake)	After correction for multiple testing (*α* = 0.003), sugars from liquid foods were positively associated with ln HOMA-IR (Q5 vs. Q1; β-coefficient, 0.11; 95% CI, 0.07–0.15; *P_trend_* < 0.001), ln-CRP (β-coefficient, 0.21; 95% CI, 0.13–0.28; *P*_trend_ < 0.001), and metabolic risk *z*-score (β-coefficient, 0.18; 95% CI, 0.13–0.24; *P*_trend_ < 0.001). No association was found for sugars from solid foods
Odegaard et al., 2010 (72)	43,580 Chinese Singaporeans (mean ± SD age, 54.8 ± 7.5 y; 42.9% males)	5 y	Consumption of soft drinks (almost never vs. 1–3 portions/mo vs. 1 portion/wk vs. 2 to ≥3 portions/wk)	Consumption of ≥2 soft drinks/wk was associated with an increased risk of T2DM (RR, 1.42; 95% CI, 1.25–1.62), compared to the lowest intake category
Palmer et al., 2008 (113)	59,000 African American females aged 21–69 y at baseline	6 y	Consumption of SSBs (<1 drink/mo vs. 1–7 drinks/mo vs. 2–6 drinks/wk vs. 1 drink/d vs. ≥2 drinks/d)	Increase in consumption was associated with increased risks of T2DM for sugar-sweetened soft drinks (*P*_trend_ = 0.002) and sugar-sweetened fruit drinks (*P*_trend_ = 0.001). Consuming ≥2 drinks/d was associated with increased risks of type 2 diabetes (incidence rate ratio, 1.24; 95% CI, 1.06–1.45) for soft drinks and fruit drinks (incidence rate ratio, 1.31; 95% CI, 1.13–1.52), compared with the lowest consumption category (<1 drink/mo)
Pacheco et al., 2022 (85)	100,314 women aged 22–104 y at baseline (median age, 53 y) from the California Teachers Study	20 y	SSB or its subtypes consumption (rare or never vs. >rare or never to <1 serving/wk vs. ≥ 1 to ≤6 servings/wk vs. ≥7 servings/wk)	For total SSBs, consumption of ≥7 servings/wk was not associated with total, CVD, or cancer mortality compared with rare or never consuming. For caloric soft drinks, a significant association was found between consumption frequency of ≥7 servings/wk and all-cause mortality (HR, 1.26; 95% CI, 1.10–1.46; *P*_trend_ = 0.02) and cancer mortality (HR, 1.33; 95% CI, 1.08–1.63; *P*_trend_ = 0.08), compared with rare or never consumption
Romaguera et al., 2013 (65)	27,058 subjects [11,684 incident cases (unknown male:female ratio) and 15,374 controls (37.8% males)] from the EPIC-InterAct study	16 y	Fruit juice and SSB consumption in glass (<1/mo vs. 1–4/mo vs. >1–6/wk vs. ≥1/d)	Higher SSB consumption was associated with higher risks of T2DM (HR for <1/mo vs. ≥1/d, 1.29; 95% CI, 1.02–1.63; *P*_trend_ = 0.013) after adjustment for confounders
				No statistically significant association between the risk of T2DM and fruit juice intake was observed (HR for <1/mo vs. ≥1/d, 1.06; 95% CI, 0.90–1.25; *P*_trend_ = 0.21) after adjustment for confounders
Schulze et al., 2004 (66)	91,249 females from the Nurses’ Health Study II aged 24–44 y at baseline	8 y	SSB consumption at baseline (<1/mo vs. 1–4/mo vs. 2–6/wk vs. ≥1/d) and change in SSB consumption between 1991–1995 (consistent ≤1/wk vs. consistent ≥1/d vs. changed from ≤1/wk to ≥1/d vs. changed from ≥1/d to ≤1/wk vs. other)	Weight gain over 4 years was higher in females who increased their consumption from ≤1/wk to ≥1/d (+4.69 kg for 1991 to 1995 and 4.20 kg for 1995 to 1999) compared with those who decreased their consumption (+1.34 and 0.15 kg for the 2 periods, respectively)Higher SSB consumption was dose-dependently associated with higher risks of T2DM (RR for <1/mo vs. ≥1/d, 1.83; 95% CI, 1.42–2.36, *P*_trend_ < 0.001) after adjustment for confounders
			
Stern et al., 2017 (69)	11,218 females from the Mexican Teachers’ Cohort (mean ± SD age, 43.3 ± 5.2 y)	2 y	Changes in consumption of sugar-sweetened soda (servings/wk): decreased (<−1) vs. no change (−1 to +1) vs. increased (>+1) vs. increase in 1 serving/d	Compared with no change, decrease in consumption by >1 serving/week was associated with less weight gain (−0.4 kg; 95% CI, −0.6 to −0.2), and increase in consumption by >1 serving/wk was associated with weight gain of 0.3 kg (95% CI, 0.2–0.5). Increase in 1 serving/d was associated with weight gain of 1.0 kg (95% CI, 0.7–1.2; *P* < 0.001)
				For change in WC, compared with no change, decrease in consumption by >1 serving/wk was associated with reduction in WC by 0.5 cm (95% CI, 0.9 to −0.1), increase in consumption by >1 serving/wk was associated with increase in WC by 0.3 cm (95% CI, 0.1–0.6). Increase in 1 serving/d was associated with change in WC by +0.9 cm (95% CI, 0.5–1.4)

1 CARDIA, Coronary Artery Risk Development in Young Adults; CHD, coronary heart disease; CRP, C-reactive protein; CVD, cardiovascular disease; E3N, The French E3N Prospective Cohort Study; EPIC, European Prospective Investigation into Cancer and Nutrition; FOS, Framingham Offspring Study; MetSyn, metabolic syndrome; NA, not applicable; NAFLD, nonalcoholic fatty liver disease; Q, quintile; SSB, sugar-sweetened beverage; T2DM, type 2 diabetes mellitus; TG, triglyceride; VAT, visceral adipose tissue; WC, waist circumference.

**TABLE 3:** Summary of observational studies examining the association between high total, free, and added sugar consumption on metabolic parameters and chronic diseases^1^

Reference, year	Subjects	Study duration	Dietary comparator	Main findings
Ahmadi-Abhari et al., 2014 (91)	25,639 adults aged 40–79 y (mean ± SD age, 61.2 ± 8.3 y, 56.5% males)	10 y	Intakes of total sugars, sucrose, and fructose	Intakes of total sugars and sucrose were not associated with risk of T2DM [HR per 40 g/d, 0.95 (95% CI, 0.83–1.08); HR per 27 g/d, 1.00 (95% CI, 0.88–1.12) for total sugars and sucrose, respectively]. Fructose intake was inversely associated with risk of T2DM (HR per 10 g/d, 0.88; 95% CI, 0.78–0.99)
Assy et al., 2008 (60)	31 patients with NAFLD (mean ± SD age, 30 ± 13 y; 53.0% males) vs. 30 healthy controls (age- and sex-matched)	NA (cross-sectional study)	Intake and sources of added sugars	Patients with NAFLD had 125% higher intake of added sugar (*P* = 0.001), and a higher proportion of their added sugar intake came from soft drink and juice (43% vs. 8%; *P* = 0.001) when compared with healthy controls, respectively
Barclay et al., 2007 (92)	4477 Australians aged 49+ y	10 y	Sugar intake (per 100 g/d)	Intake of sugar (per 100 g/d) was not associated with increased risk of T2DM [HR, 1.02 (95% CI, 0.62–1.67; *P* = 0.949) and HR, 1.09 (95% CI, 0.63–1.88; *P* = 0.767) for age- and sex-adjusted and multivariate-adjusted models, respectively]
Bergeron et al., 2021 (150)	1019 adults aged 18–65 y at baseline (50% males) from the PREDISE study	NA (cross-sectional analysis)	State of sugar-containing foods (solid vs. liquid) and form of sugar (free sugars vs. naturally occurring sugar)	High intake of free sugar from soft drinks was associated with higher fasting insulin level (1.06%; 95% CI, 0.30%–1.84%; *P* = 0.006) and HOMA-IR insulin resistance (1.01%; 95% CI, 0.19%–1.84%; *P* = 0.02) compared with the lowest intake category. Intake of naturally occurring sugar from solid foods was not associated with any outcome. After adjusting for covariates, associations were not significant for all states and forms of sugars
Burger et al., 2011 (101)	8855 males (mean ± SD age, 43.0 ± 11.0 y) and 10,753 females (42.1 ± 11.3 y) aged 21–64 y	11.9 y	Sugar intake	Sugar intake was not significantly associated with risk of CHD [HR per SD increases, 1.17 (95% CI, 0.99–1.38) and 1.10 (95% CI, 0.86–1.41) for males and females, respectively] and stroke [HRs, 1.00 (95% CI, 0.70–1.44) and 0.96 (95% CI, 0.65–1.44) for males and females, respectively]
Hodge et al., 2004 (97)	36,787 males and females aged 40–69 y without T2DM at baseline (41.0% males)	4 y	Sugar intake (per 100 g/d)	Intake of sugar (OR per 100 g/day, 0.61; 95% CI, 0.47–0.79; *P* < 0.001) was inversely associated with the incidence of T2DM. The association was weaker after adjusting for BMI and waist-to-hip ratio (OR per 100 g/d, 0.72; 95% CI, 0.56–0.93; *P* = 0.01)
Janket et al., 2003 (93)	39,345 women from the Women's Health Study (mean ± SD age, 53.3 ± 6.6 y)	6 y	Intakes of total sugar, sucrose, and fructose across quintiles	Intakes of total sugar, sucrose, and fructose were not significantly associated with increased risks of T2DM, compared with the lowest intake category [RRs, 0.86 (95% CI, 0.69–1.06), 0.84 (95% CI, 0.67–1.04), and 0.96 (95% CI, 0.78–1.19) for total sugars, sucrose, and fructose respectively]
Liu et al., 2000 (99)	75,521 women aged 38–63 y	10 y	Consumption of sucrose and fructose across quintiles	Intakes of sucrose and fructose in the highest quintile were not significantly associated with increased risks of CHD compared with the lowest quintile [RRs, 1.22 (95% CI, 0.94–1.60) and 1.07 (95% CI, 0.82–1.40) for sucrose and fructose, respectively]
Meyer et al., 2000 (78)	35,988 older Iowa women aged 55–69 y at baseline	6 y	Intakes of glucose, sucrose, and fructose across quintiles	High sucrose intake was associated with a lower incidence of T2DM (RR, 0.81; 95% CI, 0.67–0.99), compared to the lowest quintile. Higher glucose and fructose intakes were associated with increased incidences of T2DM, compared to the lowest quintile [RRs, 1.30 (95% CI, 1.08–1.57) and 0.81 (95% CI, 0.67–0.99) for glucose and fructose, respectively]
Montonen et al., 2007 (77)	4304 males and females aged 40–60 y [mean ± SD ages, 51.7 ± 8.0 y (53.8% males) and 57.5 ± 7.0 y, (37.3% males) for noncases and cases of T2DM, respectively)	5 y	Intakes of total sugar, fructose, soft drinks, and sucrose across quartiles	High total sugar intake was modestly associated with an increased incidence of T2DM (Quartile 4 vs. Quartile 1; RR, 1.56; 95% CI, 0.99–2.46; *P*_trend_ = 0.10). High fructose and soft drink intakes were associated with increased incidences of T2DM [Quartile 4 vs. Quartile 1; RRs, 1.68 (95% CI, 1.06–2.65; *P*_trend_ = 0.009) and 1.60 (95% CI, 0.93–2.76; *P*_trend_ = 0.01) for fructose and soft drinks, respectively). High sucrose intake was not associated with the incidence of T2DM (Quartile 4 vs. Quartile 1; RR, 1.22; 95% CI, 0.77–1.92; *P*_trend_ = 0.35)
Olsson et al., 2021 (86)	26,622 participants from the MDCS (39% males)	18 y	Intakes of sucrose, added sugar, SSBs, and table sugar by quintiles (percentages of daily energy intake)	Intakes of sucrose and added sugar were not associated with the risk of T2DM [Quintile 5 vs. Quintile 1; HRs, 1.03 (95% CI, 0.92–1.15; *P*_trend_ = 0.41) and 0.95 (95% CI, 0.85–1.07; *P*_trend_ = 0.65) for sucrose and added sugar, respectively]. Consumption of SSBs and table sugar were also not associated with risks of T2DM [Quintile 5 vs. Quintile 1; HRs, 1.05 (95% CI, 0.96–1.14; *P*_trend_ = 0.23) and 1.03 (95% CI, 0.93–1.14; *P*_trend_ = 0.81) for SSBs and table sugar, respectively)
Ramne et al., 2019 (79)	24,272 participants from the MDCS (mean age, 57.6 y; range, 44–73 y; 38.6% males) & 24,475 participants from the NSHDS (mean age, 48.6 y; 36–64 y; 46.3% males)	∼20 y	Intakes of free sugars and added sugars (<5% vs. 5%–7.5% vs. 7.5%–10% vs. 10%–15% vs. 15% to <20% vs. ≥20% of daily energy intake), and sugar sources (treats vs. SSBs)	Added and free sugar intakes of ≥20% of daily energy intake were associated with increased risks of all-cause mortality, compared with intake between 7.5% and <10% of daily energy intake [MDCS HR, 1.30 (95% CI, 1.12–1.51; *P* < 0.001) and NSHDS HR, 1.31 (95% CI, 1.01–1.70; *P* = 0.005) for added sugar; MDCS HR, 1.26 (95% CI, 1.10–1.44; *P* < 0.001) and NSHDS HR, 1.29 (95% CI, 1.03–1.63; *P* = 0.337) for free sugar]
				Intake of treats was inversely associated with all-cause mortality [>14 vs. ≤2 servings/wk; MDCS HR, 0.83 (95% CI, 0.74–0.93; *P* < 0.001); NSHDS HR, 0.66 (95% CI, 0.56–0.78; *P* < 0.001)], whereas intake of SSBs was positively associated with all-cause mortality [>8 vs. ≤1 servings/wk; MDCS HR, 1.14 (95% CI, 1.03–1.26; *P* = 0.035); NSHDS HR, 1.10 (95% CI, 0.90–1.35; *P* = 0.549)]
Seo et al., 2019 (87)	7005 Korean adults aged between 40–69 y (53.5% males)	NA (cross-sectional study)	Energy from total sugar intake (≤20% kcal vs. >20% kcal)	Males who consumed >20% kcal from total sugar had 49.1% (95% CI, 16.2%–91.4%), 31.3% (95% CI, 3.8%–66.0%) and 33.2% (95% CI, 3.8%–70.9%) higher odds of obesity, low HDL, and MetSyn respectively, compared with those who derived ≤20% kcal from total sugar, after adjustment for confounders. No statistically significant association between these outcomes and energy from total sugar was observed in women
Schulze et al., 2008 (94)	9702 males and 15,365 females aged 35–65 y from the EPIC- Potsdam cohort	9 ± 2 y	Intakes of sucrose and fructose across quintiles (g/d)	Intakes of sucrose and fructose were not significantly associated with risks of T2DM in men [Quintile 5 vs. Quintile 1; RRs 0.72 (95% CI, 0.50–1.04; *P* = 0.063) and 1.00 (95% CI, 0.74–1.35; *P* = 0.987) for sucrose and fructose, respectively] and women [Quintile 5 vs. Quintile 1; RRs 1.13 (95% CI, 0.74–1.74; *P* = 0.492) and 1.09 (95% CI, 0.75–1.58; *P* = 0.877) for sucrose and fructose, respectively]
Sieri et al., 2010 (100)	13,637 males (35–64 y) and 30,495 females (35–74 y)	7.9 y	Sugar intake across quartiles	Participants in the highest quartile of sugar intake did not have increased risk of CHD compared to those in the lowest quartile [RRs, 1.10 (95% CI, 0.69–1.76; *P*_trend_ = 0.83) and 0.97 (95% CI, 0.69–1.38; *P*_trend_ = 0.75) for females and males, respectively]
Sluijs et al., 2010 (98)	37,846 participants aged 21–70 y at baseline of EPIC-NL cohort (25.6% males)	10 y	Sugar intake	High sugar intake was associated with a lower incidence of T2DM (HR per SD increase, 0.87; 95% CI, 0.81–0.93; *P* < 0.001)
Sluijs et al., 2013 (95)	12,403 incident T2DM cases & 16,835 subcohort participants (37.8% males)	12 y	Sugar intake across quartiles	Sugar intake was not associated with the risk of T2DM (Quartile 4 vs. Quartile 1; HR, 0.96; 95% CI, 0.86–1.07)
Tapanee et al., 2021 (75)	524 young adults aged 18–31 y (17.4% males)	NA (cross-sectional study)	Consumption of total sugar, added sugar, sucrose, fructose, and glucose (g/d or percentage of daily energy intake)	Compared with the normal-weight controls, overweight or obese subjects had significantly higher intakes of total sugar (F_2251_ = 7.156; *P* < 0.001), added sugar (F_2251_ = 7.742; *P* < 0.001), fructose (F_2251_ = 8.243; *P* < 0.001), glucose (F_2251_ = 9.249; *P* < 0.001), and sucrose (F_2251_ = 3.592; *P* = 0.028), and higher percentage energy intakes from total sugar (F_2251_ = 11.779; *p* < 0.001) and added sugar (F_2251_ = 10.198; *P* < 0.001)
Tasevska et al., 2014 (103)	353,751 participants aged 50–71 y from the NIH-AARP Diet and Health Study (58.3% males)	13 y	Intakes of total and added sugar, total and added fructose, and total and added sucrose	No statistically significant association was observed between added sugars, total sucrose, or added sucrose for all-cause mortality or mortality from cancer, CVD, and other causes in females (Quartile 5 vs. Quartile 1)
				Compared with females with the lowest total fructose intake, those who had the highest intake had a 10% (95% CI, 4%–17%) increased risk of all-cause mortality (*P*_trend_ < 0.0001). The association was weaker in males (6% increase in risk; *P*_trend_ = 0.002)
				In males, high added sugar intake was not statistically significantly associated with the risks of all-cause mortality or mortality from cancer and CVD (Quartile 5 vs. Quartile 1). Interestingly, high added sucrose intake was found to be associated with reduced risks of CVD mortality (Quartile 5 vs. Quartile 1; HR, 0.93; 95% CI, 0.86–1.01; *P*_trend_ = 0.02)
Tasevska et al., 2018 (96)	82,254 postmenopausal women aged 50–79 y from the Women's Health Initiative Observational Study	16 y	Total sugar intake	High total sugar intake was not associated with increased risks of T2DM [HRs per 20% increase in calibrated total sugars, 0.94 (95% CI, 0.77–1.15) and 1.00 (95% CI, 0.85–1.18) in multivariable energy substitution and partition models, respectively], total CVD [HRs, 0.97 (95% CI, 0.87–1.09) and 0.91 (95% CI, 0.80–1.04)], total CHD [HRs, 0.96 (95% CI, 0.86–1.07) and 0.90 (95% CI, 0.78–1.04)], and total stroke [HRs, 1.00 (95% CI, 0.85–1.18) and 0.97 (95% CI, 0.85–1.10)].
Warfa et al., 2016 (80)	26,190 participants from the MDCS (mean ± SD age, 58 ± 7.6 y; range, 44.3–73.6 y; 38.0% males)	17 y	Sucrose intake (<5% vs. 5%–7.5% vs. 7.5%–10% vs. 10%–15% vs. >15% of daily energy intake)	Sucrose intake in the highest category (>15% of daily energy intake) was associated with an increased risk of a coronary event (HR, 1.37; 95% CI, 1.13–1.66; *P*_trend_ = 0.008), compared with the lowest intake category (<5% of daily energy intake)
Yamakawa et al., 2020 (67)	13,229 residents aged 35–69 y of Yamakawa City in Japan (44.4% males)	10 y	Intakes of free sugars, glucose, fructose, sucrose, and lactose	No statistically significant association between the intake of sugars and weight change was observed in females across quartiles
				High intakes of sucrose and fructose were associated with weight gain in males across quartiles (*P*_trends_ = 0.018 for sucrose and 0.001 for fructose)
Yang et al., 2014 (83)	11,733 adult respondents aged 20 y and older of the NHANES (48.1% males)	18 y	Percentage of daily energy intake from added sugars (0 to <9.6 vs. 9.6 to <13.1 vs. 13.1 to <16.7 vs. 16.7 to <21.3 vs. ≥21.3%)	Percentage daily energy intake from added sugar was dose-dependently associated with CVD mortality (HR for 0 to <9.6% vs. ≥21.3%, 2.03; 95% CI, 1.26–3.27; *P*_trend_ = 0.04) after adjustment for confounders

1 AARP, American Association of Retired Persons; CHD, coronary heart disease; CVD, cardiovascular disease; EPIC, European Prospective Investigation into Cancer and Nutrition; EPIC-NL, European Prospective Investigation into Cancer and Nutrition–Netherlands; MDCS, Malmö Diet and Cancer Study; MetSyn, metabolic syndrome; NA, not applicable; NAFLD, nonalcoholic fatty liver disease; NSHDS, Northern Swedish Health and Disease Study; PREDISE, Rédicteurs Individuels, Sociaux et Environnementaux; SSB, sugar-sweetened beverage; T2DM, type 2 diabetes mellitus.

Several studies suggest that high consumption of SSBs may be positively associated with the risks of obesity and related complications (12, 59–75), such as nonalcoholic fatty liver disease (NAFLD) (60, 64), T2DM (61–63, 65, 72, 76–80), stroke (71), coronary heart disease (CHD) (71, 81, 82), high blood pressure (15, 70, 73), and CVD mortality (83–85). However, in the prospective cohort study by Olsson et al. (86), high intake of SSBs was not associated with an increased risk of T2DM. Other studies reported that high SSB consumption was associated with MetSyn in men but not women, which may be explained by differential hormone levels between males and females (81, 82, 87). Furthermore, a recent review of prospective cohort studies and short-term trials (13) suggests that regular consumption of SSBs was associated with hyperuricemia and gout, which could further increase the risks for T2DM, CVD, and MetSyn, in addition to dyslipidemia (13, 88), inflammation (13, 89), and decreased leptin (90). Nonetheless, its relationship with stroke is somewhat inconclusive (12).

Several prospective cohort studies have shown that high sucrose or fructose consumption was not associated with the T2DM incidence or risks (86, 91–96), nor was it even associated with a reduced risk of T2DM (78, 91, 97, 98) (Table 3). In contrast, Warfa et al. (80) showed in a prospective cohort study that high sucrose consumption was associated with an increased risk of T2DM, and the study by Montonen et al. (77) showed high fructose intake, but not sucrose intake, was associated with an increased T2DM incidence. For cardiometabolic health, studies (77, 78, 86, 91, 93, 94, 96, 99–101) have shown that high intakes of both sucrose or fructose and total sugars were not associated with increased risks of total CVD, total CHD, or total stroke. Results were inconsistent for CVD or all-cause mortality, with some studies suggesting an adverse association between added sugar and mortality (79, 102), while Tasevska et al. (103) reported null findings in women or even a protective effect in men.

These observed associations could be due to both direct (unique metabolic changes induced by fructose, such as increased hepatic DNL without inducing weight gain) and indirect (promotion of weight gain and obesity, leading to adverse metabolic effects) effects of fructose (22). In a prospective cohort study conducted in an Asian population (*n* = 43,580) (72), high soft drink consumption was associated with increased risk of T2DM, independently of changes in BMI, and weight gain in addition to high soft drink consumption exerted an additive effect on increasing risk of T2DM. Similarly, regular SSB consumption was associated with higher C-reactive protein (CRP) levels (104), and this association was strengthened by obesity (89), whereas sugars from solid foods were not associated with increased CRP levels (104). In another prospective cohort study conducted by Tasevska et al. (103) (*n* = 353,751), high total fructose but not added sugar consumption in both males and females was found to be related to a modest increase in the all-cause mortality risk. This was only restricted to fructose in SSBs, not fructose present in solid foods, which is in line with the conclusions by Togo et al. (16) and DiMeglio and Mattes (15). However, high intake of free or added sugars was found to be positively associated with all-cause mortality in the prospective cohort study conducted by Ramne et al. (79). Moreover, high consumption of solid sugar sources was inversely associated with all-cause mortality, and high intake of SSBs was positively associated with all-cause mortality (79). Similarly, in the prospective cohort study (*n* = 25,877) by Janzi et al. (71), while high added sugar intake was associated with increased risks of coronary events and stroke and high SSB consumption was associated with an increased risk of stroke, low added sugar intake was found to increase the risks of aortic stenosis and atrial fibrillation and low consumption of sugar-sweetened solid foods increased the risks of stroke, coronary events, and atrial fibrillation. All these studies support the differential effects of liquid compared with solid forms of carbohydrates in inducing overeating and obesity (liquid > solid). High SSB consumption may also be implicated in the pathogenesis of NAFLD, as shown in a cross-sectional study (*n* = 73) (105), in addition to being implicated in hypertriglyceridemia (prospective cohort study; *n* = 2774) (70).

Overall, the findings from observational studies remain inconclusive. Our views agree with a previous systematic review of prospective cohort studies (106), which concluded that high SSB consumption increases the risk of cardiovascular diseases both directly and indirectly through weight gain. Additionally, in a recent meta-analysis of 11 prospective cohort studies that assessed the associations between SSB intake and risks of CVD and mortality (102), long-term consumption of SSBs was dose-dependently associated with increased risks of CVD morbidity and mortality. Similarly, in another meta-analysis of 24 observational studies (12 longitudinal studies, 11 cross-sectional studies, and 1 case-control study) (107), high SSB intake was associated with an increased risk of MetSyn compared to low SSB consumption. The meta-analysis of 11 prospective cohort studies also showed that participants in the highest quantile of SSB consumption (most often 1–2 servings/day) had greater risks of developing T2DM and MetSyn compared to those in the lowest quantile of SSB consumption (0 or <1 serving/month) (108). However, high SSB consumption was only associated with the development of MetSyn in a pooled analysis of cross-sectional studies, not in prospective cohort studies, in the systematic review and meta-analysis of 8 cross-sectional and 4 prospective cohort studies by Narain et al. (109). This discrepancy might be caused by the relatively low sample size compared with previous meta-analyses. The meta-analysis and systematic review of 22 prospective cohort studies and 10 clinical trials conducted by Malik et al. (8) suggests that long-term consumption of SSBs is linked to weight gain in both children and adults. It should be noted that all these systematic reviews and meta-analyses only examined added sugar in the liquid form (i.e., SSBs), but not sugar in the solid form. Moreover, all observational studies mentioned in this review supporting weight gain in humans after high sugar consumption focused on SSBs (12–14), which may have a stronger obesogenic effect, as the liquid form of added or free sugars may not be able to induce the compensatory calorie saving that the solid form of sugars could (19). A recent systematic review and meta-analysis of 13 prospective cohort studies (*n* = 49,591) conducted by Azad et al. (110) examined the associations of both solid and liquid sources of sugar with incidences of MetSyn, and found that while high SSB consumption was linked to an increased incidence of MetSyn (with a moderate certainty of evidence), solid sources of sugar, including ice cream and confectionaries, were not associated with incident MetSyn, although the certainty of evidence was very low. Thus, it is possible that sugar in the solid form does not produce comparable health impacts to SSBs, and more evidence is needed to address this question. Additionally, it was suggested that high sugar consumption is linked to obesity and metabolic diseases due to the provision of excess calories, not the role of sugar itself (7, 111–113). Individuals who consume a diet high in sugars often have other unhealthy dietary and lifestyle habits, such as a lack of exercise, high fat intake, and smoking, all of which could contribute to the pathogenesis of obesity-related disorders (109, 114).

#### Evidence from clinical trials

Similar to observational studies, there is also inconsistency in the conclusions from human clinical trials, which may be due to different study designs (**Tables 4** and **5**). Some studies report that high SSB intake could increase the risks for chronic diseases, such as T2DM, CVD, obesity, hyperglycemia, dyslipidemia, and ectopic fat accumulation (115–129). Such increases in disease risks were commonly believed to be due to the excess energy contributed by sugars, rather than the unique effect of sugar intake per se. For example, high consumption of SSBs results in increased energy intake and weight gain, overweight, or obesity (*n* = 41) (120), whereas reduction of SSB intake leads to higher weight loss (116, 117), possibly in a dose-dependent manner (116). In another intervention study involving 71 abdominally obese men and lasting for 12 weeks, the researchers found that consumption of moderate amount of fructose (75 g of fructose per day in the form of beverages) led to significant yet small increases in body weight and waist circumference, as well as increases in the liver fat content. Although the authors did not find any association between changes in energy intake and weight gain, this is likely due to measurement errors or insufficient statistical power, as the statistically nonsignificant increase in daily energy intake (i.e., 54 kcal/day × 84 days = 4536 kcal) should translate into ∼2.5 kg of weight gain (vs. the 1.1 kg reported) (129). Similarly, in a randomized, double-blind study by Johnston et al. (130), during the isocaloric period, high fructose intake (25% daily energy intake) in the form of liquids did not increase weight and liver fat accumulation and disrupt liver function compared to the control group (25% daily energy intake from glucose) in healthy but overweight men. Nevertheless, when on a hypercaloric diet, both high fructose and glucose intake led to similar increases in body weight, liver fat, and biomarkers of liver function.

**TABLE 4:** Summary of clinical trials examining the effects of high SSB consumption on metabolic health^1^

Reference, year	Subjects	Study duration	Intervention	Main findings
Aeberli et al., 2011 (125)	29 healthy young males (mean ± SD age, 26.3 ± 6.6 y)	Six 3-wk interventions separated by a minimum of a 4-wk washout period	600 mL SSBs per day containing 40 g fructose (medium fructose; 6.5% daily caloric intake) vs. 80 g fructose (high fructose; 13% daily caloric intake) vs. 40 g glucose (medium glucose) vs. 80 g glucose (high glucose) vs. 80 g sucrose (high sucrose) vs. dietary advice to consume low amounts of fructose	Mean ± SD waist-to-hip ratio was significantly higher in all interventions containing fructose (0.92 ± 0.05 to 0.93 ± 0.05) compared to baseline (0.92 ± 0.06; *P* < 0.0083)LDL particle size was significantly smaller in high-fructose (mean ± SD, −0.51 ± 0.80) and high-sucrose interventions (mean ± SD, −0.43 ± 0.81) compared with baseline. There was a significant decrease in large LDL I subclasses in the medium-fructose, high-fructose, and high-sucrose groups (*P* < 0.0083)Fasting glucose and CRP rose significantly after all interventions (by 4%–9% and 60%–109% respectively; *P* < 0.05)
Bruun et al., 2015 (124)	47 overweight but otherwise healthy subjects (mean ± SEM age, 38.6 ± 1.1 y; 36.2% males)	6 mo	1 L of sugar-sweetened cola, aspartame sweetener cola, semi-skimmed milk, or still mineral water	Only those in the sugar-sweetened cola group had an increase in serum uric acid level at the end of the intervention (15% increase; *P* = 0.02)
				No significant change in body weight or total fat mass was observed in all groups, but the sugar-sweetened cola group had a significant increase in VAT of 30% (*P* = 0.02), and a more than 2-fold increase in hepatic fat (*P* = 0.01)
Ebbeling et al., 2006 (116)	103 adolescents aged between 13–18 y who were regular SSB consumers (mean ± SD ages, 16.0 ± 1.1 vs. 15.8 ± 1.1 for intervention and control groups, respectively; 44.3% males)	25 wk	Weekly home deliveries of noncaloric beverages vs. control (consumption of SSB)	Those who received weekly home deliveries of noncaloric beverages had a lower increase in BMI compared with controls, although the difference was statistically nonsignificantA subgroup analysis revealed a significant difference between the intervention and control groups only amongst those with baseline BMIs ≥ 25.6 kg/m^2^ (mean ± SEM changes in BMI, −0.63 ± 0.23 kg/m^2^ vs. +0.12 ± 0.26 kg/m^2^; *P* = 0.03)
Geidl-Flueck et al., 2021 (127)	94 healthy, young males aged 18–30 y	7 wk	Beverages sweetened with 80 g/d of fructose, sucrose, or glucose vs. control (nonconsumption)	Compared with the control group, consumption of beverages sweetened with fructose and sucrose led to a 2-fold increase in basal hepatic fractional secretion rates [median FSR percentages per day: sucrose, 20.8 (*P* = 0.0015); fructose, 19.7 (*P* = 0.013); control, 9.1], whereas glucose had no significant effect on FSR
				Compared to the control, absolute secretion rates of newly synthesized VLDL palmitate was increased after consumption of fructose-sweetened beverages (*P* = 0.055) and sucrose-sweetened beverages (*P* = 0.008)
Hieronimus et al., 2020 (134)	145 healthy young adults aged 18–40 y (49.0% females)	2 wk	Beverages sweetened with aspartame (noncaloric control) vs. 25% daily caloric intake from glucose vs. 17.5% or 25% kcal from fructose vs. 10%, 17.5% or 25% kcal from HFCS vs. 25% kcal from sucrose	Compared with the control group, a 24-hour increase in TG level was highest after consuming beverages sweetened with 25% daily energy intake from fructose (6.66 mmol/L × 24 hours; 95% CI, 1.90–11.63; *P* = 0.0013), increase in levels of LDL cholesterol and apoB were highest after consuming beverages sweetened with 25 daily energy intake kcal from HFCS [0.46 mmol/L (95% CI, 0.16–0.77; *P* = 0.0002) and 0.108 g/L (95% CI, 0.032–0.184; *P* = 0.001), respectively]
James et al., 2004 (117)	644 children aged 7–11 y (mean ± SD age, 8.7 ± 0.9 y; 49.7% girls)	1 school y	School-based focused nutrition education program aimed at reducing SSB consumption vs. control (no intervention)	The intervention results in a decrease in SSB consumption by 0.6 glasses, which correlates with a 0.2% point decrease in the proportion of overweight and obese children. This is in contrast to the increase in both measures in the control group
Johnston et al., 2013 (130)	31 overweight but otherwise healthy males aged 18–50 y	2-wk isocaloric period +6-wk washout period +2-wk hypercaloric period	High fructose vs. glucose intake in the form of beverages (25% of daily calories)	During the isocaloric period, both high-fructose and high-glucose intake led to stable body weight, liver TG, and concentrations of liver enzymes, including ALT and AST, and the intergroup difference was not significant
				During the hypercaloric period, both interventions led to similar increases in body weight, liver TG, and concentrations of ALT and AST
Low et al., 2018 (118)	16 healthy adults (mean ± SEM ages, 42.8 ± 1.8 vs. 46.6 ± 0.9 for males and females, respectively; 50% males)	2 study d separated by a 4-wk washout period	Low- fructose (20 g) vs. high-fructose (60 g) drinks	Significantly higher contribution of DNL fatty acids to VLDL-TG after high fructose consumption (time × meal interaction *P* < 0.01). No significant difference was observed in males
Maersk et al., 2012 (119)	47 overweight but otherwise healthy subjects aged 20–50 y (63.8% females)	6 mo	1 L of sugar-sweetened cola, aspartame sweetener cola, semi-skimmed milk, or still mineral water	Sugar-sweetened cola resulted in significantly higher liver fat, skeletal muscle fat, visceral fat, blood TG, and total cholesterol than the other beverages. However, no significant difference was observed for total fat mass
Pearson et al., 2021 (126)	8 young healthy males (22 ± 1.79 y)	1 d per diet separated by a 1-wk washout period (cross-over design)	Mixed macronutrient meal with 20 oz of diet coke (artificially sweetened) or regular coke (HFCS sweetened) or control (water)	Sugar-sweetened cola resulted in significantly lower fat oxidation and higher carbohydrate oxidation than artificially sweetened cola (*P* = 0.006 and 0.014, respectively) and water (*P* = 0.001 and 0.001, respectively)
Raben et al., 2002 (120)	41 overweight males and females (mean ± SEM ages, 33.3 ± 2.0 vs. 37.1 ± 2.2 in high- and low-sucrose groups, respectively; 14.6% males)	10 wk	152 vs. 0 g/d sucrose supplements (∼70% from beverages and ∼30% from solid foods)	Sucrose supplements, mostly in the form of beverages, resulted in significant increases in energy intake (+1.6 MJ/d; *P*_diet×time_ = 0.03), body weight (+1.6 kg; *P*_diet×time_ < 0.0001), fat mass (+1.3 kg; *P*_diet×time_ < 0.05), and systolic and diastolic blood pressure (+3.8 and 4.1 mmHg, respectively)
Sigala et al., 2020 (128)	131 adults aged 18–40 y (51.9% males)	2 wk	Beverages sweetened with aspartame or 25% energy requirement as glucose, fructose, HFCS, or sucrose	There was no significant difference in body weight change between groups.High-sucrose (+14%; *P* < 0.0015), high-fructose (+9%; *P* = 0.015), and HFCS (+8%; *P* = 0.017) intakes increased energy intake compared with the aspartame group (−4%; *P* = 0.0037).High-fructose intake decreased 24-hour leptin AUC (−13.6 ± 7.6 ng/ml × 24 hours; *P* = 0.0008) compared with sucrose
Stanhope et al., 2009 (122)	32 overweight and obese subjects (50% males)	10 wk	25% kcal daily kcal requirement from glucose- vs. fructose-sweetened beverages	The fructose group but not the glucose group had a significant increase in VAT, despite similar weight gain in both groups
				DNL and postprandial TG were both higher in the fructose group, which coincided with increases in markers of dyslipidemia, such as apoB and LDL, as well as insulin resistance
Stanhope et al., 2015 (121)	85 adults (aged 18–40 y; 49.4% males)	20 d	Artificially sweetened beverages vs. SSBs providing 10 vs. 17.5 vs. 25% daily kcal requirement	Compared with the artificially sweetened beverages, the HFCS-containing SSBs caused increases in postprandial TG, as well as increased fasting and postprandial LDL cholesterol, apoB and apoCII, and uric acid
Taskinen et al., 2017 (129)	71 abdominally obese men (mean ± SD age, 49.1 ± 10 y; range, 21–65 y)	12 wk	Beverages sweetened with 75 g/d of fructose, no control group (pretest vs. post-test)	Fructose consumption significantly increased the liver fat content (mean ± SD, +0.67 ± 2.2%; *P* = 0.008). There were also significant but minor increases in body weight (mean ± SD, +1.1 ± 1.7%; *P* < 0.0001) and waist circumference (mean ± SD, +0.67 ± 2.5%; *P* = 0.006)

1 ALT, alanine aminotransferase; AST, aspartate aminotransferase; CRP, C-reactive protein; DNL, de novo lipogenesis; FSR, fractional secretion rate; HFCS, high-fructose corn syrup; SSB, sugar-sweetened beverage; TG, triglyceride; VAT, visceral adipose tissue.

**TABLE 5:** Summary of clinical trials examining the effects of high total, free, and added sugar consumption on metabolic health^1^

Reference	Subjects	Study duration	Intervention	Main findings
Bantle et al., 2000 (115)	24 healthy adults (50% males)	6 wk on each diet (cross-over design)	17% daily energy intake from fructose vs. 17% daily energy intake from glucose	32% higher day-long plasma TG concentration in males at the end of the fructose diet period than that in the glucose diet period (*P* < 0.001). No similar effect was observed in females
Black et al., 2006 (133)	13 healthy male subjects (mean ± SEM age, 33 ± 3 y)	6-wk diet separated by a 4-wk washout	Low-sucrose (10% daily energy intake) vs. high-sucrose (25% daily energy intake) diet	There was no significant difference in body weight, fasting plasma glucose, fasting serum insulin, total, LDL cholesterol and TG levels, or blood pressure between groups. However, the high-sucrose group had significantly higher LDL (mean ± SEM, 2.78 ± 0.30 vs. 2.25 ± 0.25 mmol/L, respectively; *P* < 0.01) and total cholesterol (mean ± SEM, 4.62 ± 0.8 vs. 4.01 ± 0.80 mmol/L, respectively; *P* < 0.01) levels than the control group
Bravo et al., 2013 (131)	80 adults (mean ± SD age, 42.2 ± 11.7 y; 56.3% males)	10 wk	Sucrose or HFCS at 8%, 18%, or 30% daily energy intake required for weight maintenance	No significant difference between sucrose vs. HFCS treatment in the liver or muscle fat
Lewis et al., 2012 (135)	13 overweight or obese but otherwise healthy adults (mean ± SEM age, 46.1 ± 1.9 y; 69.2% males)	Two 6-wk dietary periods separated by a 4-wk washout	Low-sucrose (5% daily caloric intake) vs. high-sucrose (15% daily energy intake) diet	There was no significant difference in body weight or composition, peripheral glucose utilization, lipid profiles, blood pressure, or vascular compliance between groups. However, fasting glucose was significantly higher after the high-sucrose diet compared to the control (mean ± SEM, 5.4 ± 0.2 vs. 5.0 ± 0.2 mmol/L, respectively; *P* < 0.01)

1 HFCS, high-fructose corn syrup; TG, triglyceride.

In contrast, results from some studies suggested that the effects of sugar on these health outcomes were independent of the excess energy contributed by the sugar. For example, a double-blind, randomized controlled trial in 94 healthy men reported that consumption of SSBs containing moderate amounts of fructose or sucrose (80 g/day) increased fatty acid synthesis in the liver even in the basal state, compared to the control group (nonconsumption), without inducing weight gain (127).

Results from studies examining the effects of high sugar consumption on circulating lipids and fat accumulation were also inconsistent. High SSB consumption was found to increase blood TG levels, as well as ectopic fat accumulation in the liver, muscle, and viscera (115, 119, 122–124, 129). Sex differences in such effects were also reported in a cross-over trial (*n* = 16) (118). In contrast, other studies failed to detect a persistent effect of high SSB intake on fasting plasma concentrations of cholesterol, HDL cholesterol, and LDL cholesterol, in both males and females (*n* = 24) (115), as well as of ectopic lipid accumulation in the liver and muscle (*n* = 80) (131). It is worth noting that in the latter study, the investigators added different amounts of fructose and HFCS to low-fat milk, which on its own has been shown to benefit cardiometabolic health (132), thus potentially confounding the results. The randomized cross-over trial conducted by Black et al. (133) (*n* = 13) in healthy subjects also found no significant difference between low-sucrose (10% daily energy intake) and high-sucrose (25% daily energy intake) diets (from both solid and liquid foods) on body weight, insulin sensitivity, fasting plasma glucose and serum insulin, and blood pressure. However, the high-sucrose group had significantly higher total and LDL cholesterol levels than the control group (133). The study by Hieronimus et al. (134) (*n* = 145) showed that high fructose consumption for 2 weeks led to the greatest increase in TG compared to HFCS, glucose, and aspartame (*P* = 0.0013 vs. aspartame), and high HFCS consumption led to the greatest increase in LDL cholesterol and apoB compared to fructose, glucose, and aspartame (*P* = 0.0002 and 0.001, respectively, vs. aspartame). A post hoc assessment found that there was a significant interaction between glucose and fructose in contributing to the significant increases in levels of LDL cholesterol and apoB, but not TG. However, this study has several limitations that affected the validity of conclusions. First, the statistical analysis only compared HFCS and fructose with aspartame, not other types of added or free sugars. Aspartame is a noncaloric, artificial sweetener that is used to replace sugar in foods and beverages, so we are not sure whether HFCS or fructose would also lead to significantly higher increases in cardiovascular risk factors compared to other types of sugars. Second, all sugars examined in this study exist in liquid form, which, as discussed earlier, may have differential impacts on health outcomes. Third, this study lasted only 2 weeks, so it is not known whether the observed effect was going to last over the long term.

Mixed results have also been reported for the effects of high sugar consumption on the macronutrient metabolism. In a randomized, cross-over study conducted in healthy, young males (*n* = 29) (125), the authors showed that even 6.5% and 13% of daily energy intake consumption of fructose and sucrose, respectively, from SSBs could impair the carbohydrate and lipid metabolisms. However, this study was limited by the short study duration of only 3 weeks and a possible carry-over effect of previous interventions throughout the study. Similarly, moderate (about 13% daily energy intake) consumption of a cola soft drink ingested as part of a mixed meal decreased fat and increased carbohydrate oxidation compared to the control drink (water) (126). However, this study was also limited by a small sample size (*n* = 8). Lewis et al. (135) compared the effects of high-sucrose (15% daily energy intake) and low-sucrose (5% daily energy intake) diets (from both solid and liquid foods) on body compositions and outcomes of carbohydrate and lipid metabolisms in overweight or obese subjects who were already moderately insulin resistant (*n* = 13). Their results indicate that there were no differences in body weight, body composition, insulin resistance, lipid profiles, or blood pressure between groups, except the fasting blood glucose level, which was significantly lower in the low-sucrose diet group. In contrast, conclusions from the meta-analysis of 38 randomized controlled trials conducted by Schwingshackl et al. (136) suggest that isocaloric replacement of fructose and sucrose with starch could lead to lower LDL cholesterol levels, insulin resistance, and lower uric acid levels, further adding controversies.

Overall, high SSB intake may increase the risks of T2DM and CVD via induction of hyperglycemia or glucose intolerance and of dyslipidemia due to increased DNL (118, 129), circulating TG, VLDL (118, 121), and uric acid (5, 124). Also, high consumption of fructose-sweetened beverages may disrupt the production of appetite control hormones (decreases in leptin and insulin and increases in ghrelin; *n* = 12) (123, 128), supporting the differential effects of liquid compared with solid sugars on metabolic and endocrine health.

#### Limitations of clinical trials

Several important limitations exist which curtail the validity of conclusions. First, similar to animal studies, most clinical trials are conducted over a short period, which rarely lasts longer than 6 to 8 weeks, although it is acknowledged that subjecting participants to high sugar intake for a longer period may be unethical and impractical, as it is difficult for study participants to adhere to a dietary intervention for a longer period. Second, glucose or fructose alone is used in some studies; however, in real life, they usually coexist in foods (e.g., in HFCS). It has also been pointed out that studies comparing the effects of HFCS with other sweeteners are limited (120). Third, the energy balance is not controlled in some trials; hence, it is impossible to discern whether the observed effects were due to intake of sugar per se or to excessive caloric intake. Fourth, similar to animal studies, many clinical trials examine doses of sugars that are higher than normal human consumption, which is not necessarily realistic and does not lend support to the current guidelines to restrict free sugar intake to below 10% of the daily energy intake. Finally, some clinical trials involved subjects who were overweight or obese or were already hyperglycemic or insulin resistant. Thus, evidence linking high sugar intake with increased risks for chronic diseases comes in part from those who were more susceptible to these diseases, and may not apply to healthy individuals.

### Issues with sugar reformulation programs and potential consequences of government policies directed towards reducing sugar intake

We argue that the current public health recommendations to encourage the reduction of both solid and liquid forms of free sugar intake (e.g., sugar reformulation programs that set targets for both solid and liquid foods) should be revised due to the overextrapolation of the results from SSB studies. Moreover, there are other important issues associated with the implementation and effectiveness of sugar reformulation programs. First, sugar has important functional properties in food that other sweeteners cannot completely replace, such as flavor enhancement, color formation, bulk and texture, fermentation, and preservation (137). Second, there are challenges associated with labeling of added sugars, as added sugars cannot be differentiated from total sugars chemically (137) and there is no universal definition for added sugars (138). However, this may not pose a problem for manufacturers, who have the exact formulation of their products. Third, when sugar is removed from a food product, the bulk and texture of the product is usually affected, and bulking agents such as modified starch are commonly utilized to solve the issue. However, these agents generally provide energy because they are carbohydrate-based. As a result, eventually the caloric content could even increase compared to the original formulation (137).

## Discussion

While it seems to be a consensus among researchers and public health practitioners that high free sugar consumption, regardless of the sources, is associated with ill health (2, 3), in our opinion the substantial limitations in the current body of evidence, especially from animal studies, such as short study durations, the use of supraphysiological doses of sugar or fructose alone, and the lack of appropriate controls, seriously curtail the translatability of the findings to the real-world situation. More studies should also be conducted to further confirm whether free sugars in solid and liquid forms exert similar adverse effects on health. Such studies should be conducted over a longer-term period (at least 6 months) with added and free sugar intakes that better resemble the human diet (20%–25% of daily energy intake). In animal studies that examine the underlying mechanisms of the effects of sugar intake, a lower-sugar diet (e.g., 10% daily energy intake from added or free sugars) should be used as the control diet to better reflect human consumption patterns.

In all, we think the current guidelines on reducing free sugar intake to prevent weight gain and obesity are based on low-quality evidence (7) that requires cautious interpretation by policy-makers and the general public. While some may argue that a high-sugar diet is usually more nutrient dilute (139, 140), newer analyses (141–143) suggest an extremely low–sugar diet (<5% daily energy intake) may also have similar nutrient-diluting effects. This is likely because some sugar-rich foods and beverages are indeed a good source of nutrients, such as breakfast cereals. Indeed, sugar may improve the palatability of nutrient-rich foods, such as rolled oats, that are otherwise bland to consume on their own. It is quite possible that “high” sugar consumption at normal dietary doses (e.g., 25% of the daily energy intake), especially in the solid form, may not be uniquely obesogenic or harmful for health. Therefore, the public health emphasis should be on restricting the intakes of specific energy-dense, nutrient-poor high-sugar foods, such as cakes and biscuits, rather than limiting sugar intake from all foods. To date, many countries have implemented taxes on SSBs. While this has been effective in reducing SSB consumption, whether the tax is also effective in preventing obesity and cardiometabolic diseases is still questionable based on the major limitations in the current body of evidence (144). Also, although low-calorie artificial sweeteners provide significantly less energy than sugars and have been widely used in food products as an alternative to sugars (145, 146), a number of studies have shown that these sugar substitutes could cause weight gain, cancers, and side effects, and more well-designed, large-scale human studies on the health effects of low-calorie artificial sweeteners are needed in the future (147).

## Notes

The authors reported no funding received for this study.

Abbreviations used: CHD, coronary heart disease; CRP, C-reactive protein; CVD, cardiovascular disease; DNL, de novo lipogenesis; GI, glycemic index; HFCS, high-fructose corn syrup; MetSyn, metabolic syndrome; NAFLD, nonalcoholic fatty liver disease; SSB, sugar-sweetened beverage; T2DM, type 2 diabetes mellitus; TG, triglyceride; wt:wt, weight:weight.
